# Genome Maps, a new generation genome browser

**DOI:** 10.1093/nar/gkt530

**Published:** 2013-06-08

**Authors:** Ignacio Medina, Francisco Salavert, Rubén Sanchez, Alejandro de Maria, Roberto Alonso, Pablo Escobar, Marta Bleda, Joaquín Dopazo

**Affiliations:** ^1^Department of Computational Genomics, Centro de Investigación Príncipe Felipe (CIPF), Valencia 46012, Spain, ^2^CIBER de Enfermedades Raras (CIBERER), Valencia 46012, Spain, ^3^Genometra S.L., Valencia, Spain and ^4^Functional Genomics Node (INB) at CIPF, Valencia 46012, Spain

## Abstract

Genome browsers have gained importance as more genomes and related genomic information become available. However, the increase of information brought about by new generation sequencing technologies is, at the same time, causing a subtle but continuous decrease in the efficiency of conventional genome browsers. Here, we present Genome Maps, a genome browser that implements an innovative model of data transfer and management. The program uses highly efficient technologies from the new HTML5 standard, such as scalable vector graphics, that optimize workloads at both server and client sides and ensure future scalability. Thus, data management and representation are entirely carried out by the browser, without the need of any Java Applet, Flash or other plug-in technology installation. Relevant biological data on genes, transcripts, exons, regulatory features, single-nucleotide polymorphisms, karyotype and so forth, are imported from web services and are available as tracks. In addition, several DAS servers are already included in Genome Maps. As a novelty, this web-based genome browser allows the local upload of huge genomic data files (e.g. VCF or BAM) that can be dynamically visualized in real time at the client side, thus facilitating the management of medical data affected by privacy restrictions. Finally, Genome Maps can easily be integrated in any web application by including only a few lines of code. Genome Maps is an open source collaborative initiative available in the GitHub repository (https://github.com/compbio-bigdata-viz/genome-maps). Genome Maps is available at: http://www.genomemaps.org.

## INTRODUCTION

Genome browsers are extremely useful to represent, compare and correlate information from different sources [that include single-nucleotide polymorphisms (SNPs), gene expression, methylation, chromosomal alterations and so forth] on a genomic context (1). Genome browsers are mainly used in two scenarios: visualization of user’s genomic data and visualization of genomic features, which is typically associated to updates of genome projects. Given the sizes of data files, which range from several megabytes of the files of processed genomic data (e.g. GFF or VCF) to >50 GB of intermediate genomic data (e.g. exome BAM files, with sizes >200 GB in low-coverage genomes), current solutions for their visualization are local. Thus, the most popular BAM viewer, the IGV (2), must be installed locally, and, to our knowledge, no web-based solutions for the visualization of local BAM or VCF files are currently available.

On the other hand, if the aim is the visualization of genome features, different genome browsers on the web are available, with Ensembl (3) and UCSC (4) being the most popular ones. This popularity comes mainly from the amount of information they serve, given their character of front end of repositories of genome projects. However, with the continuous increase in the available genomic data and metadata along with the limitations derived from extensive data traffic imposed by client/server architecture, these browsers are becoming slower and less efficient in terms of usability. New web-based browsers use more advanced web technologies, such as Ajax (Asynchronous JavaScript and XML) technology (5–7), which provide more dynamic interfaces. Nevertheless, they are far from being able to offer the amount of information that the genome repositories can do. Moreover, many current web-based genome browsers are implemented using images that are processed in the server and sent to the browsers, like GBrowse (8), Ensembl (3) or UCSC (4), which results in slow responses to requests from the client. To overcome this problem, other web-based genome browsers, such as XMap (9), follow a similar approach to Google Maps and break images into tiles that are downloaded to the browser as needed. In all cases, images and files can be dynamically loaded or updated in the client-side using Ajax technology. However, sending images over the network always constitutes a bottleneck. A partial exception to this rule of not taking advantage of the enormous interactivity offered by modern web browsers is JBrowse (6) that sends part of the data as static cached files to the client that renders this information using HTML4 elements.

Here, we present Genome Maps, which follows a different approach to traditional genome browsers and takes advantage of new HTML5 standard, specially the scalable vector graphics (SVG) inline and the FileReader API. The use of these technologies makes it unnecessary to send images to the client-side or to install any Java Applet, Flash plug-in or any other plug-in technology and results in a fast and dynamic response to user requests. Genome Maps allows real-time navigation along chromosomes and karyotypes, representing different types of data over many types of genomic information. Moreover, to our knowledge, this is the only web-based solution that allows users to upload local large BAM or VCF files and navigate through them dynamically in the client side. As the use of massive sequencing is expanding to clinic, where data are subjected to privacy laws, the possibility of keeping sensitive files locally is a desirable property of any genome browser. Genome Maps represents a new generation of efficient genome viewers in which many heterogeneous genomic data and metadata can be integrated and displayed in the web browser with no need of any installation or update.

## MATERIALS AND METHODS

### Browser functionality

#### The browser framework

**Figure 1.**
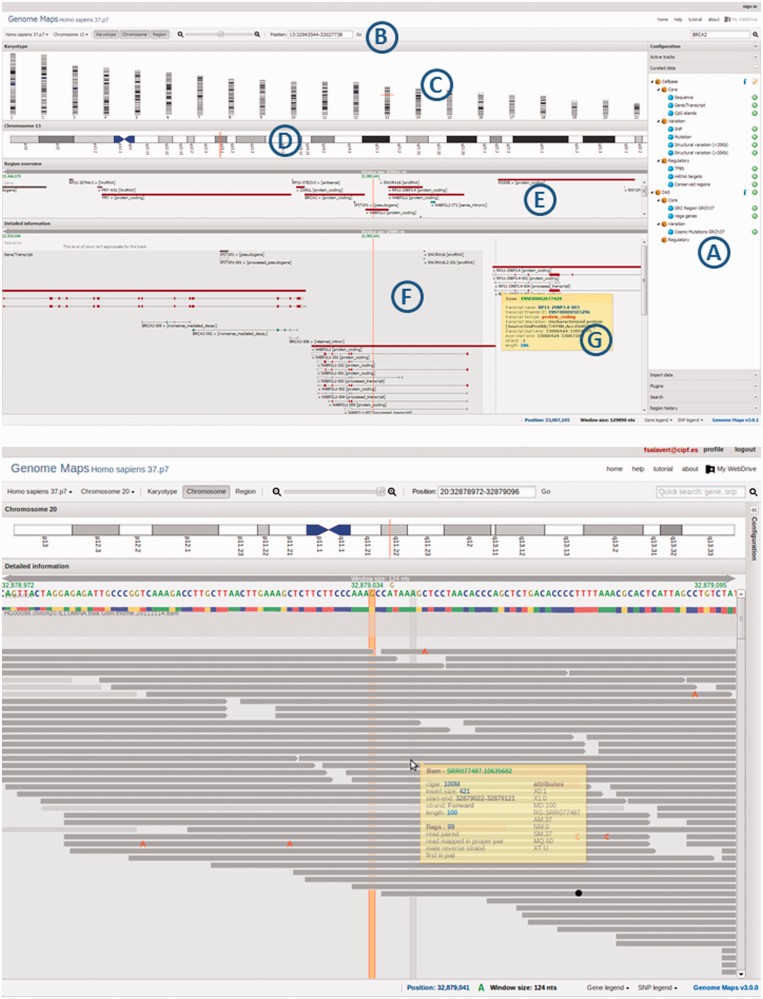
The upper part of the figure represents a screenshot of the Genome Maps interface showing all the information provided by the browser. In the lateral collapsible menu bar (**A**) tracks or DAS servers can be activated, also plug-ins are found there. In the navigation bar (**B**) the controls to move to different genomes, zoom or locations are found. ‘Karyotype’ (**C**), ‘Chromosome’ (**D**) and ‘Region Overview’ (**E**) panels show different resolution levels of information. The ‘Detailed information’ (**F**) panel displays all the tracks. By default sequence, genes and SNPs tracks are activated. Setting the cursor over an element pops up a window with relevant information (**G**). The lower part of the figure represents another screenshot of Genome Maps with a BAM file with the zoom at base resolution detail. The status bar shows some useful information such as genomic position or color legends. Here, the right-hand menu bar has been hidden.

As seen in Figure 1 (top), the web interface contains the conventional elements of a genome browser. Genome annotations are represented within the context of the corresponding reference genome, with an overhead navigation bar giving a visual indication of the chromosome coordinates displayed in the view. Navigation is done by dragging the display left or right, which produces a smooth panning effect. The zoom level can be changed to have different views at different details (from the highest level, at nucleotide resolution, to lowest levels). The view configuration can easily be changed through a menu that allows adding or removing different tracks with different types of genomic information (see later in the text). Many gene features are available for genes, transcripts and exons, which include their different types of variations, functional annotations and regulatory elements. A DAS client has also been implemented. If the cursor is set over any element in the view, a small window pops up with all the relevant information on that element (Figure 1G). There is also a search function that can use all the information aforementioned, giving a broad range of search possibilities.

#### Navigation bar and menu

This bar allows users to select the species genome and the chromosome to visualize (Figure 1B). Three buttons activate the ‘Karyotype’, ‘Chromosome’ and ‘Region overview’ panels (Figure 1C–E, respectively). A slider allows for the selection of zoom levels from sequence level to whole chromosome view. Users can move to specific locations by either introducing the chromosome and position or writing the beginning of the name of the gene or transcript (>20 different gene IDs are available).

On the right side, there is a menu with the active tracks, the features and DAS servers available, the search option and the facilities to data importation (including the upload of local files).

#### Tracks

Genome Maps can show a great variety of information using different tracks. Three tracks are visible by default: (i) at highest zoom level a sequence track shows the genome sequence, (ii) genes, transcripts, exons and introns are showed at high levels in the next track, these are colored according to their biotype and (iii) SNPs are shown in another track and colored according to their consequence type. A pop-up window displays useful information when the mouse is set over them. When a feature name is clicked, then a window is shown with comprehensive information on it. For example, if a transcript is clicked, a window is displayed with detailed information on its exons, SNPs, mutations, transcription factor-binding sites (TFBSs), miRNA targets and so forth. At lower zoom levels, there are too many features to be visualized, and a histogram showing feature density is displayed instead. Tracks can be rearranged to accommodate user preferences. Many other tracks can be activated from the menu, which include CpG island, mutations, structural variations, TFBS or miRNA targets among others. Genes and transcripts colors are displayed according to their biotype meanwhile for SNPs, the consequence type is used. Gene and SNPs legends are shown at the bottom part.

Moreover, a DAS client has been developed to display information from either pre-configured DAS from the menu or adding new external DAS servers into Genome Maps.

#### User data input

A novel capability of Genome Maps is the possibility of importing local files to the browser and further representing it in the corresponding genome context. Genomic data in different standard formats (GFF, BED, VCF and BAM) can easily be imported from local disk or from a remote server provided by Genome Maps and displayed as tracks on the viewer. To our knowledge, this extremely useful feature is, among the current web-based genome browsers, unique to Genome Maps.

### Information and databases

Biological information is taken from CellBase (10). Apart from genome sequences and the coordinates of genes, transcripts and exons, CellBase serves more information on variants, including SNPs (11), cancer-related mutations (12), HapMap genotype frequencies (13) and structural variations (14). Functional information from different sources, including GO (15), Interpro (16) and Reactome (17) is also available. Also, regulatory information, including Jaspar (18) TFBSs, CpG islands, binding sites for histones, polymerases, open chromatin regions and conserved regions, taken from Ensembl (14) and miRNA targets (19), is accessible in Genome Maps. The ChemDoodle library (http://web.chemdoodle.com/) has been used to represent the 3D structures of the proteins, taken from PDB, when available. Protein sequence, variants and features are taken form UniProt (20). This information is exported in JSON format through a Java RESTful web service API. The genomes of 16 vertebrates (including 5 primates), 5 metazoan, 6 fungi, 3 protists and 7 plants, which include the most common model organisms, are currently available in the browser. Reference genomes are taken from Ensembl (14).

### Architecture design and performance

Genome Maps has been developed as a fast and efficient Javascript fully browser-side application; therefore, no installation or updates are required. The program uses the new web technology HTML5 and the SVG; therefore, graphics are provided without resorting to proprietary software plug-ins. The connection between the client browser and the server database has been designed to increase the usability by obtaining only the information requested by the user that fits in the screen. To provide a real-time navigation, the coming regions are pre-fetched, and data are rendered in the background before user moves into them. Data are sent compressed to the client to optimize bandwidth usage and stored in a local Javascript cache system.

**Figure 2.**
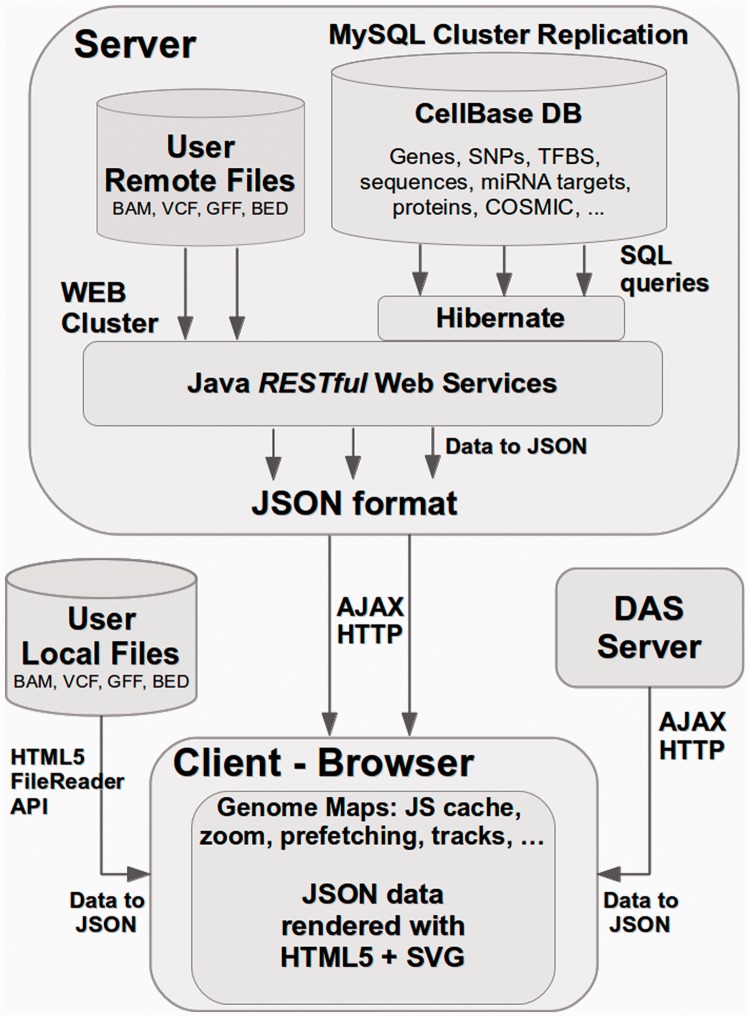
Schema of the client-server architecture of Genome Maps and the technologies used. The user navigates along the genome and requests different types of information. This produces queries to the remote server that through a RESTful web service manages the queries to the database and serves the requested information. DAS servers can also serve information on Genome Maps. Users can also visualize different types of data (in different formats such as BAM, VCF, BED or GFF) on the genomic context. In all cases, data are converted to JSON format. These data are used to render SVG objects into DOM. JSON data are stored in a Javascript cache system in the client.

Genome Maps architecture design takes an innovative approach to render features in the browser. As seen in Figure 2, data can be obtained from different sources. Genome Maps can query CellBase web services from the server, and JSON data are rendered as HTML5 SVG objects into the DOM (Document Object Model, the primary data structure used by a web browser to represent an HTML page, see http://www.w3.org/DOM/) directly using Ajax technology. A custom SVG API was developed to render JSON data. JQuery 1.8.3 is used to manage DOM. Sencha Ext JS 4.1 is used to implement user interfaces. Also, HTML5 FileReader API is used to read user local files. JSON parsers were developed for the different formats. JSON data are stored in an efficient and fast Javascript cache developed for Genome Maps. This cache allows minimizing the remote connections and permits a fast access to stored data. Data files uploaded by users (BAM, VCF and so forth) are automatically indexed either at the client side for local uploads or at server side if the data are imported to the server, to optimize queries. Data are also exported in JSON format. Data can be also imported either from DAS servers or from user local or remote files. These data are converted to JSON format before being rendered as SVG elements.

At the server side, data are hosted in a MySQL 5.1 Cluster Replication with a high cache to allow fast data retrieval (Figure 2). To provide a high availability and load balancing to the MySQL replication cluster, a Keepalived (http://www.keepalived.org) and HAProxy (http://haproxy.1wt.eu/) services have been configured. To achieve a higher performance, Java was chosen for the server implementation to both (i) connect to MySQL using JBoss Hibernate (http://www.hibernate.org) and (ii) to implement RESTful web services API using Jersey library (http://jersey.java.net). Apache Tomcat 6 was chosen as Java application server to deploy the web archive (war file) with the RESTful web services API implementation. To provide a high availability and load balancing to these web services a HAProxy was set-up to balance Apache Tomcat instances. This configuration provides an efficient and high-availability solution with no single point of failure. Sessions with login have also been implemented. In logged sessions, the data imported in the server are maintained for further logins.

This approach, which has proven to be efficient and provides a real-time navigation, has many benefits: (i) server side is light, as Java only executes SQL queries to a MySQL cluster server, which have been optimized to respond in <5 ms, (ii) traffic of data is minimized, as data sent in JSON format are GZipped before to be sent to the client, (iii) SQL queries and Java RESTful web services are easily cached in the server, (iv) HTML5 SVG standard is rich in events and allows easy interaction with the genome browser, (v) as data are rendered in the client-side from a JSON format, users can convert to JSON local files with features in GFF or VCF format and load them into the web-based genome browser and (vi) new data can be added to or embedded in the browser in different projects in a straightforward way, given that only a JSON format is needed to render the features.

Performance has been studied in several common workstations and browsers (Google Chrome 20+, Apple Safari 5+, Opera 11+, Internet Explorer 10 and Mozilla Firefox 16+). In general, the results were excellent, rendering thousands of features in <1 s even with a standard dual-core with 4 GB of random access memory. However, we are working to improve the performance in Internet Explorer and Mozilla Firefox that, although pretty good, it was not at the level of the other browsers’.

To show a smooth panning effect and avoid latencies because of weak computers or low-internet bandwidth, an intelligent pre-fetching system has been developed. Genome Maps does the rendering of the coming region before user moves into there. These optimizations make Genome Maps efficient and fast, providing users with a real-time web-based genome browser.

As an additional option, server side code is distributed in a web archive (WAR) that can be downloaded to be installed in user’s resources to export their genomes and data.

Also, a public Amazon machine image (AMI) has been configured with server side code and Genome Maps client application. Users can launch this image in Amazon AWS and start using their own Genome Maps instance without the need of any installation or set-up.

### Open and collaborative project

Genome Maps has been released as an open project under GPLv2 license, and its future development is open to the developers’ community. The code, including the Genome Maps server application that exports data in a highly optimized and efficient way, is available in the GitHub repository: https://github.com/compbio-bigdata-viz/genome-maps.

### Other projects using genome maps

Genome Maps has been designed to be easily embedded in other bioinformatics projects. Thus, a Javascript API has been designed for developers to manipulate every aspect of Genome Maps such as the species or tracks to be shown with just a few Javascript calls. Integrating a fully operating version of Genome Maps in any web application is as easy as including in the HTML the two lines below after importing the libraries:

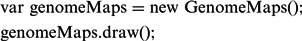



A proof of the simplicity of this implementation is the VARIANT (21) project, which contains an instance of Genome Maps to show the annotated genomic variants from a VCF file. Genome Maps has also been integrated in other projects such as the PatSeqExplorer (http://www.lens.org/lens/bio/patseqexplorer) to display portions of the genome resulting from a search.

## DISCUSSION

Genome browsers are of increasing importance as more genomes are sequenced. However, this upsurge of information is, at the same time, causing a subtle but continuous decrease in the efficiency of conventional genome browsers. Moreover, the widespread use of next-generation sequencing technologies is generating an increasing need for representing user’s genomic data over the genomic layouts, an option offered only by some local genome browsers to date.

Genome browsers that are gateways to genome projects such as the genome browser at UCSC (4) or Ensembl (3) are often rich in information but their performances are not optimal. Some alternatives are available, such as the WebGBrowse (22), that offer a web interface to the popular stand-alone GBrowse (8) and others (23). Different solutions adopting new technologies that overcome many performance problems have been proposed. Thus, JBrowse (6), the NCBI viewer (http://www.ncbi.nlm.nih.gov/projects/sviewer/) or Genome Projector (5) use Javascript, Ajax and SVG. Actually, SVG is starting to be used by recently released genome browsers such as the Scribl (24), published while this manuscript was submitted, and the not yet published Genoverse (http://www.genoverse.org/). Other genome browsers like Dalliance (7) also use DAS technologies. Despite some web-based browsers like Ensembl or UCSC offer the possibility of importing user’s data files, this must be done via public URLs. This requires from end users to have free access to an internet web server, which is unusual, as well as a high-network bandwidth to upload big files. Genome Maps can also import files, and does it directly, without the need of a URL. However, unlike current web-based genome browsers, Genome Maps also offers the possibility of loading and visualizing locally user’s BAM or VCF files, avoiding thus data transfer from the client to the server. This possibility is only offered by stand-alone applications (2,25–27), but they either require the installation of large databases (with the inherent complications associated) or experience the problems of internet limitations when third party databases need to be queried. This is an extremely useful feature when data security is an issue, given that all sensitive genomic data can be kept on the user’s side without the requirement of moving them through internet.

The fast growing databases of genomes and related information must be queried in the process of visualization by genome browsers. Accordingly, the dilemma is either having the data locally together with a stand-alone application, or serving a few data to a client-side browser with enough visualization capability. Following a philosophy similar to Google, here we choose an innovative solution: heavy data are not moved through the web. The information needed at each moment by the genome browser is requested to a remote server that queries the database on the server side via a RESTful web service and returns the response to the client. The resulting query process is extremely fast. A main collateral advantage of this scenario is that no installation or updated of databases are needed. The database with the required information is always up-to-date in the remote server. Genome Maps offers also capabilities only found on stand-alone applications, such as the possibility of importing huge local files of genomic data (e.g. VCF or BAM) and dynamically displaying them on the proper genomic context. In particular, this property is extremely useful in any genome browser used in clinic given the privacy laws that affect genomic data and impose restrictions on the movement of such data.

In conclusion, Genome Maps includes the newest web technologies, such as HTML5, with Javascript and SVG, which makes of it an extremely efficient client-side application. Genome Maps offers the better of the two worlds: the ease of implementation and use of a web browser (no installation or update requirements) and the performance of a stand-alone genome browser (with the possibility of uploading and visualizing local files of genomic user’s data offered for the first time by a web-based genome browser). Genome Maps constitutes a new generation tool for genomic data visualization that changes internally and functionally the conventional genome browser’s way of operating.
